# Synthesis and Fungicidal Activities of (*Z*/*E*)-3,7-Dimethyl-2,6-octadienamide and Its 6,7-Epoxy Analogues

**DOI:** 10.3390/molecules201219743

**Published:** 2015-11-25

**Authors:** Mingyan Yang, Hongbo Dong, Jiazhen Jiang, Mingan Wang

**Affiliations:** Department of Applied Chemistry, China Agricultural University, Beijing 100193, China; yangmy@cau.edu.cn (M.Y.); dhb1986115@cau.edu.cn (H.D.); jiangjiazhen@cau.edu.cn (J.J.)

**Keywords:** 3,7-dimethyl-2,6-octadienamide, 3,7-dmethyl-6,7-epoxy-2-octadienamide, synthesis, fungicidal activity

## Abstract

In order to find new lead compounds with high fungicidal activity, (*Z*/*E*)-3,7-dimethyl-2,6-octadienoic acids were synthesized via selective two-step oxidation using the commercially available geraniol/nerol as raw materials. Twenty-eight different (*Z*/*E*)-3,7-dimethyl-2,6-octadienamide derivatives were prepared by reactions of (*Z*/*E*)-carboxylic acid with various aromatic and aliphatic amines, followed by oxidation of peroxyacetic acid to afford their 6,7-epoxy analogues. All of the compounds were characterized by HR-ESI-MS and ^1^H-NMR spectral data. The preliminary bioassays showed that some of these compounds exhibited good fungicidal activities against *Rhizoctonia solani* (*R. solani*) at a concentration of 50 µg/mL. For example, **5C**, **5I** and **6b** had 94.0%, 93.4% and 91.5% inhibition rates against *R. solani*, respectively. Compound **5f** displayed EC_50_ values of 4.3 and 9.7 µM against *Fusahum graminearum* and *R. Solani*, respectively.

## 1. Introduction

**Scheme 1:**
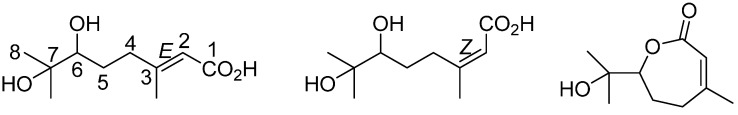
Structures of (*Z*/*E*)-3,7-dimethyl-2,6-octadienoic acid and its 1,6-olide.

**Scheme 2:**
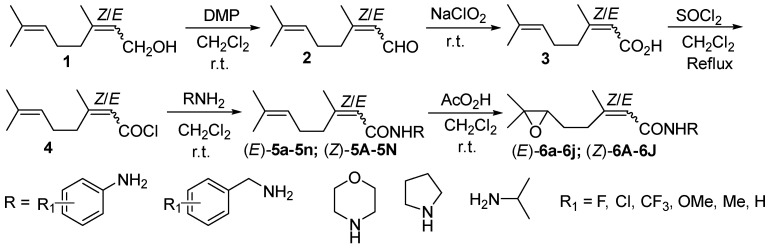
Synthetic route of (*Z*/*E*)-3,7-dimethyl-2,6-octadienamide and their 6,7-epoxy analogues.

Amide compounds were widely used in pharmaceutical and agrochemical fields due to their wide range of biological activity. In pharmaceutical chemistry, some amides showed potent antibacterial activities and antiproliferative against human cancer cell lines including the drug-resistant cancer cells [1,2,3]. The other amides not only induce a significant decrease of antibiotic resistance in Gram-negative bacteria [4], but also exhibit antimicrobial activity against *Staphylococcus aureus* and *Bacillus subtilis* [5,6,7]. In agricultural chemicals, a lot of novel amide derivatives have been synthesized, some of them showed good fungicidal or insecticidal activities, and the mode of action on amide fungicides has been reviewed recently [8,9,10,11,12,13,14,15,16]. 2,6-Dimethyl-6-hydroxy-2*E*,4*E*-hepta-2, 4-diene acid and (*6R*)-3,7-dimethyl-7-hydroxyl-2-octen-1,6-olide were isolated from the fruit of *Litsea cubeba* in Tibet, and they were evaluated to have good fungicidal activities in our laboratory [17,18]. Based on these results, some of the seven-membered lactone derivatives were synthesized and confirmed to exhibit moderate to excellent fungicidal activities [19,20,21]. To the best of our knowledge, the biological activities of monoterpene acid amides were seldom paid attention. In order to find some novel derivatives with excellent fungicidal activity and explore the differences between 3,7-dimethyl-2,6-octadienoic acid and 3,7-dimethyl-7-hydroxyl-2-octen-1,6-olide (Scheme 1) against phytopathogens, (*Z*/*E*)-3,7-dimethyl-2,6-octadienoic acids were synthesized via two-step selective oxidation with the commercial available nerol/geraniol as the starting material [19,22,23]. Then, 28 different (*Z*/*E*)-3,7-dimethyl-2,6-octadienamide derivatives were designed and synthesized by reaction of the acid chloride and various aromatic and aliphatic amines [24], and their 6,7-epoxy derivatives were further obtained by epoxidation of the double bond between C-6 and C-7 [25]. The fungicidal activities of these amides and their 6,7-epoxy analogues against *Fusahum graminearum*, *Rhizoctonia solani*, *Alternaria solani*, *Sclerotinia sclerotiorum*, and *Botrytis cinerea* were evaluated. The synthetic route is shown as below (Scheme 2).

## 2. Results and Discussion

As we know, (*Z*)-3,7-dimethyl-2,6-octadienoic acid and (*6R*)-3,7-dimethyl-7-hydroxyl-2-octen-1,6-olide exhibited certain fungicidal activities [17,18]. The racemic 3,7-dimethyl-7-hydroxyl-2-octen-1,6-olide, the other seven-membered lactone derivatives and (*E*)-3,7-dimethyl-2,6-octadienoic acid were synthesized and some of them were found to exhibit better fungicidal activities in our laboratory [19,20,21]. In the total synthesis, we found that (*E*)-6,7-dihydroxy-3,7-dimethyl-2,6-octadienoic acid is an unstable compound because it is easily dehydrated under acid condition that needs to be purified rapidly and kept in low temperature. In this case, the 6,7-epoxy moiety was designed to replace the 6,7-dihydroxy group and keep the stability of the compound. Then 3,7-dimethyl-6,7-epoxy-2-octadienamides were designed and synthesized to observe the effect of epoxy moiety on the fungicidal activities. In consideration of the instability of epoxy moiety in strong acid condition, the route strategy of epoxidation-amidation was abandoned due to low product yields, the approach of amidation-epoxidation was selected to avoid the side reaction after optimizing repeatedly the reaction condition. The key intermediates (*Z*/*E*)-3,7-dimethyl-2,6-octadienoic acid were easily prepared in 85% and 76% yields over two steps with the Dess-Martin oxidant and Pinnick oxidation utilizing the commercially available nerol/geraniol as raw material [19]. Finally, the amides and the 6,7-epoxy amide derivatives were afforded in 15%–79% yields and 63%–96% yields under the mild condition, respectively.

In the ^1^H-NMR of compounds **5a**–**5n**, the olefin protons on C-2 exhibited a singlet with the chemical shifts δ 5.47–5.77, the olefin protons on C-6 displayed a multiplet at δ 4.90–5.20 due to the coupling with the adjacent methylene protons at C-5 and long range coupling with CH_3_ at C-7. The methyls on C-3 had a doublet at δ 2.23–2.05 with the coupling constant 1.2 Hz due to the long range coupling with the proton on C-2, and the amide protons had the broad singlet with the chemical shifts δ 5.27–7.57. While for the *cis* isomers **5A**–**5N**, the amide protons had the similar chemical shifts as the *trans* isomers, but the chemical shift of the protons on C-2 and C-6 shifted to the downfield about δ 0.02–0.10, and the methyls on C-3 shifted to the upfield about δ 0.30–0.40.

In the ^1^H-NMR of 6,7-epoxy compounds **6a**–**6j**, the protons on C-2, the amide protons and the methyls on C-3 also showed the similar chemical shifts and coupling constants as that of compounds **5a**–**5n**. However, the protons on C-6 had the chemical shifts δ 2.53–2.76 due to existence of 6,7-epoxy group and the peaks split into the double of doublet due to the coupling with the two protons at C-5 with the coupling constants 5.5 and 7.0 Hz, the chemical shifts of the two methyls at C-7 shifted to upfield about δ 0.30–0.35. Similarly, for the *cis* isomers **6A**–**6J**, the protons on C-2, the protons on C-6, the amide protons, the methyls on C-3, and the two methyls at C-7 had the similar chemical shifts and coupling constants. All of the new compounds were also characterized by HR-ESI-MS, and the [M + H]^+^ peaks were detected; their exact mass numbers matched well with the calculated molecule weights.

Based on the data in Table 1, compounds **5** and **6** showed a broad-spectrum of fungicidal activities against five tested agriculturally important phytopathgens; they were found to be particularly active against *Rhizoctonia solani* and *Alternaria solani*, for example, **5C**, **5I** and **6b**, respectively, exhibited 94.0%, 93.4% and 91.5% inhibition rates against *R. solani*, while **5a**, **5c**, **5d**, **5g**, **5I** and **5M** showed 86.4%, 86.0%, 88.9%, 88.7%, 89.5%, and 85.5% inhibition rates against *A. solani* at the concentration of 50 µg/mL, respectively. Greater than 70% inhibition at 50 µg/mL was considered to be good in terms of antifungal inhibition, greater than 90% excellent in this paper.

In the *Z*/*E*-amides, comparison of the inhibition rates of **5l**, **5m**, **5n**, **5L**, **5M** and **5N** with **5a**–**5k** and **5A**–**5K**, we found that the aromatic amides showed much better fungicidal activities than the aliphatic amides against *R. solani* and *A. solani.* It seemed that the aromatic substituted group contributed a lot to the fungicidal activities. Thus, the different aromatic groups such as phenyl, substituted phenyl, benzyl and substituted benzyl groups were selected to optimize the structure. By comparing the inhibition rates of compounds **5b**–**5g**, **5B**–**5G** with compound **5a** and **5A**, we found that the *ortho*-substitution (Cl, F) was beneficial to improve the fungicidal activities such as **5c**, **5d**, **5C** and **5D**, while the activities at the *para*-substitution were kept or reduced such as **5b**, **5e**, **5f**, **5B**, **5e** and **5F**. However, one more *N*-methyl group did not significantly change the activity comparing **5a** and **5A** with **5g** and **5G**. So we concluded that the *para*-substitution was not helpful to improve the activity, especially the electron-withdraw substitution groups. Further, the amides with benzyl and substituted benzyl groups (compounds **5h**–**5k** and **5H**–**5K**) were synthesized and assayed. The results in Table 1 indicated that amides with (substituted) benzyl groups had similar or increased fungicidal activities against *R. solani* and *A. solani* comparing with compound **5a** and **5A**. Thus, the (substituted) benzyl groups have similar effects on the fungicidal activities as the (substituted) phenyl groups. The effect of the double bond configuration at C_2_ and C_3_ on the inhibition rates did not indicate significant differences by comparison the inhibition data of **5a**–**5**n and **5A**–**5N**.

From the data in Table 1, it was very clear that compounds **5** showed much better fungicidal activities against all tested phytopathgens than compounds **6**. While compounds **6b** and **6B** were two exceptions, which showed the inhibition rates of 91.5% and 82.7% against *R. solani*, respectively, much higher than the 59.5% and 55.4% inhibition rates of **5b** and **5B**. The similar effects were observed for the aromatic and aliphatic amides, the substitution on the benzene ring of phenyl and benzyl groups, and the configuration of the double bond at C_2_ and C_3_. The double bond at C_6_ and C_7_ or adjacent 6,7-dihydroxy played an essential role for a better fungicidal activity when comparison the inhibition rate data of compounds **5** and **6**.

**Figure 1:**
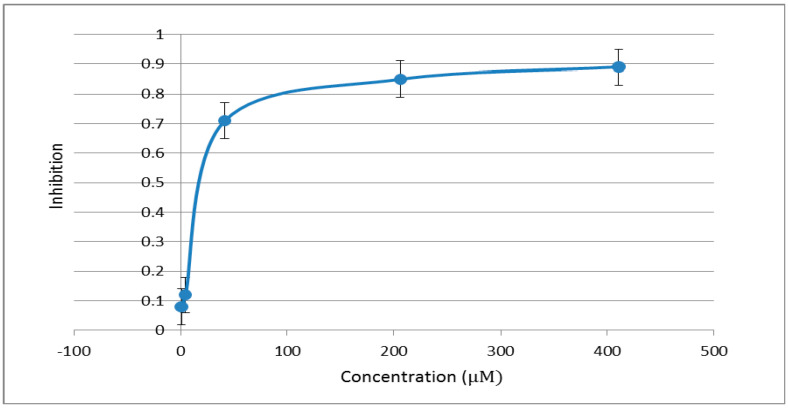
Inhibition rates of **5a** against *A. solani* at different concentrations.

Based on the above results, the EC_50_ values (EC_50_ is the concentration of inhibition 50% fungus growth at tested condition.) were determined further for these compounds with more than 70% inhibition rates. The typical inhibition rates changing with the concentration could be seen in Figure 1. The data in Table 2 confirmed that most of compounds exhibited an inhibition against *R. solani* and *A. solani* with EC_50_ values between 9.7 and 677.8 µM, and several compounds were active against *F. graminearum*, *S. sclerotiorum* and *B. cinerea* with EC_50_ values between 4.3 and 92.9 µM. Among them, compound **5f** had the best fungicidal activities with EC_50_ values of 4.3 and 9.7 µM against *F. graminearum* and *R. solani*, respectively, and compounds **5g** and **5I** had the broad-spectrum of fungicidal activities against four phytopathgens with EC_50_ values between 17.1 and 61.2 µM, most of the other compounds have EC_50_ values between 13.4 and 97.9 µM against *R. solani* and *A. solani* except **6d**, **6f**, **5M** and **6H**. These results indicated that there would be the possible improvement of fungicidal activities against *R. solani* and *A. solani* if the chemical structures were further modified, especially on the structures of **5f**, **5g** and **5I**. Optimizations on the aromatic amine moieties around compound **5** are in progress.

**Table 1:** The fungicidal activities (inhibition rate, %) of compounds **5** and **6** at 50 µg/mL ^a^.

Compd.	R	*F. G*	*R. S*	*A. S*	*S. S*	*B. C*	Compd.	R	*F. G*	*R. S*	*A. S*	*S. S*	*B. C*
**5a**	Ph	68.1	88.9	86.4	62.9	60.2	**5A**	Ph	63.7	79.9	85.0	72.3	66.7
**5b**	2,4-Cl_2_Ph	17.7	59.5	77.6	36.2	22.9	**5B**	2,4-Cl_2_Ph	47.2	55.4	51.3	56.5	4.6
**5c**	2-ClPh	51.9	85.3	86.0	57.4	44.4	**5C**	2-ClPh	52.2	94.0	81.3	63.4	44.5
**5d**	2-FPh	74.3	84.3	88.9	47.9	48.0	**5D**	2-FPh	55.2	82.3	82.4	45.3	43.5
**5e**	4-CF_3_Ph	73.4	65.6	63.9	23.4	26.8	**5E**	4-CF_3_Ph	55.0	83.8	70.5	52.5	31.2
**5f**	4-CH_3_Ph	77.0	86.0	77.1	53.6	26.5	**5F**	4-CH_3_Ph	46.7	71.4	74.4	70.4	42.7
**5g**	Ph, CH_3_	50.6	86.7	88.7	77.1	83.2	**5G**	Ph, CH_3_	71.3	86.4	85.1	69.2	65.4
**5h**	PhCH_2_	55.5	86.7	72.4	34.6	51.0	**5H**	PhCH_2_	53.6	80.7	80.2	57.0	34.6
**5i**	4-FPhCH_2_	30.7	54.4	82.0	24.7	31.6	**5I**	4-FPhCH_2_	75.2	93.4	89.5	76.9	47.4
**5j**	4-OCH_3_PhCH_2_	45.3	83.0	78.9	46.6	64.0	**5J**	4-OCH_3_PhCH_2_	52.5	77.6	81.6	46.9	19.1
**5k**	2-ClPhCH_2_	44.5	79.9	81.1	69.5	64.6	**5K**	2-ClPhCH_2_	56.6	89.3	83.3	69.7	51.0
**5l**	morpholino	16.4	44.8	50.9	40.7	17.8	**5L**	morpholino	30.1	44.9	62.2	14.2	7.3
**5m**	pyrrolidin-1-yl	39.7	60.0	79.8	28.1	29.2	**5M**	pyrrolidin-1-yl	63.4	78.8	85.5	43.1	35.6
**5n**	isopropyl	15.6	48.7	58.2	12.8	14.3	**5N**	isopropyl	46.2	50.3	43.2	43.1	26.0
**6a**	Ph	39.1	23.2	38.0	19.2	16.9	**6A**	Ph	32.8	30.3	69.3	36.4	22.8
**6b**	2,4-Cl_2_Ph	63.0	91.5	73.6	72.7	30.4	**6B**	2,4-Cl_2_Ph	46.9	82.6	81.1	43.7	39.1
**6c**	2-ClPh	39.8	35.6	59.1	33.3	13.5	**6C**	2-ClPh	33.2	30.4	15.4	33.9	31.6
**6d**	4-CH_3_Ph	74.0	79.2	58.4	18.4	32.9	**6D**	4-CH_3_Ph	46.7	43.9	73.4	3.72	2.50
**6e**	Ph, CH_3_	68.5	59.1	66.5	46.2	10.7	**6E**	Ph, CH_3_	36.2	62.6	54.2	0.0	1.6
**6f**	PhCH_2_	65.0	57.7	71.3	0.0	9.7	**6F**	PhCH_2_	54.3	76.8	57.8	31.7	19.8
**6g**	4-FPhCH_2_	17.7	68.4	29.1	0.0	15.1	**6G**	4-FPhCH_2_	40.4	38.9	55.7	28.7	10.9
**6h**	morpholino	68.6	52.5	43.3	38.7	33.2	**6H**	morpholino	33.8	73.3	52.9	0.0	17.6
**6i**	pyrrolidin-1-yl	9.2	37.8	66.3	19.9	0.0	**6I**	pyrrolidin-1-yl	59.3	48.1	76.3	0.0	12.1
**6j**	isopropyl	22.6	18.0	65.4	8.4	15.2	**6J**	isopropyl	36.3	53.0	36.8	10.6	38.4
Carbendazim		100	100	100	81.0	4.2	Chlorothalonil		95.8	99.9	100	100	100

^a^ *F. G*: *Fusahum graminearum*, *R. S*: *Rhizoctonia solani*, *A. S*: *Alternaria solani*, *S. S*: *Sclerotinia sclerotiorum*, *B. C*: *Botrytis cinerea*. The data are the mean measurements were calculated from the three replicates with 0 ± 5% errors.

**Table 2:** The EC_50_ (µM) values with 95% confidential interval in parenthesis of compounds **5** and **6** against different phytopathgens for the compounds with more than 70% inhibition rates in Table 1.

Compd.	*F. graminearum*	*R. solani*	*A. solani*	*S. sclerotiorum*	*B. cinerea*
**5a**	-	26.1 (20.3–33.6)	19.8 (14.5–27.1)	-	-
**5b**	-	-	51.0 (45.3–57.4)	-	-
**5c**	-	33.9 (26.0–44.0)	28.6 (25.8–31.7)	-	-
**5d**	-	19.2 (12.7–25.6)	39.8 (28.6–48.6)	202.7 (155.0–263.1)	-
**5e**	11.9 (7.6–16.9)	-	-	-	-
**5f**	4.3 (3.2–5.8)	9.7 (25.1–56.2)	37.6 (45.6–59.5)	-	-
**5g**	-	19.1 (13.9–26.1)	27.2 (21.9–33.7)	61.2 (44.5–101.2)	56.2 (38.2–82.6)
**5h**	-	30.4 (24.5–37.6)	27.6 (21.3-35.7)	-	-
**5i**	-	-	32.8 (25.5–42.1)	-	-
**5j**	-	35.5 (25.2-44.4)	27.8 (23.3–29.6)	-	-
**5k**	-	29.8 (24.1-32.9)	41.3 (32.0–47.5)	-	-
**5m**	-	-	50.3 (33.3–75.7)	-	-
**6b**	-	62.3 (44.9-86.6)	43.4 (31.1–60.5)	21.6 (13.8–33.5)	-
**6d**	57.4 (52.7–62.3)	130.7 (92.1–185.1)	-	-	-
**6f**	-	-	189.4 (155.4–230.4)	-	-
**5A**	-	97.9 (88.3–108.4)	14.0 (11.5–17.1)	92.9 (67.9–126.9)	-
**5C**	-	80.1 (56.4–113.4)	62.6 (41.8–93.6)	-	-
**5D**	-	54.5 (35.6–73.5)	26.0 (19.7–30.2)	-	-
**5E**	-	51.2 (42.5–55.5)	22.9 (16.9–27.9)	-	-
**5F**	-	39.0 (29.5–51.4)	42.6 (30.6–59.0)	23.2 (16.8–31.9)	-
**5G**	-	13.4 (11.6–15.5)	35.9 (27.0–47.6)	125.2 (96.3–161.3)	-
**5H**	-	41.4 (26.8–63.6)	21.2 (16.3–27.4)	-	-
**5I**	38.8 (32.5–46.3)	18.7 (12.6–27.6)	17.1 (12.6–23.2)	40.7 (35.2–47.0)	-
**5J**	-	57.9 (38.9–76.7)	31.1 (23.5–36.6)	-	-
**5K**	-	18.6 (14.9–20.8)	32.0 (25.3–36.1)	-	-
**5M**	-	284.1(207.7–387.9)	63.8 (42.6–95.3)	-	-
**6B**	-	31.6 (20.3–49.2)	43.4 (28.8–65.1)	-	-
**6D**	-	-	667.3 (462.3–999.5)	-	-
**6F**	-	>1000	-	-	-
**6H**	-	677.8 (622.7–736.6)	-	-	-
**6I**	-	-	83.8 (56.9–123.1)	-	-

## 3. Experimental Section

### 3.1. General Information

All reactions were performed with magnetic stirring. Unless otherwise stated, all reagents were purchased from commercial suppliers and used without further purification. Organic solutions were concentrated under reduced pressure using a rotary evaporator or oil pump. Melting points were measured on a Yanagimoto apparatus (Yanagimoto MFG Co., Kyoto, Japan) and uncorrected. ^1^H-NMR spectra were obtained on Bruker DPX 300 spectrometer (Bruker Biospin Co., Stuttgart, Germany) with CDCl_3_ as a solvent and TMS as an internal standard. High-resolution mass spectral analysis was performed on a LTQ Orbitrap instrument (ThermoFisher scientific Inc., Waltham, MA, USA).

### 3.2. Synthesis

#### 3.2.1. Synthesis of (*Z*/*E*)-3,7-Dimethyl-2,6-octadienal (**2**, Neral and Geranial)

According to the literature protocol [19,22], geraniol (8.0 g, 52 mmol), Dess-Martin Periodinane (26.6 g, 63 mmol) and 320 mL DCM were added to a 500 mL three-necked flask at room temperature. The mixture was stirred for 4 h at the room temperature. Then the mixture was filtered and the filtrate was washed with saturated NaHCO_3_ solution, brine and dried over anhydrous Na_2_SO_4_. The solvent was removed under reduced pressure. The residue was purified by column chromatography using silica gel (petroleum ether/EtOAc 15:1) to give (*E*)-3,7-dimethyl-2,6-octadienal (7.9 g, 94%) as a colorless oil; the (*Z*)-isomer (10.7 g, 88%) was also prepared as a colorless oil using nerol (12.3 g) as starting material following the same procedure [26,27].

#### 3.2.2. Synthesis of (*Z*/*E*)-3,7-Dimethyl-2,6-octadienoic Acid (**3**, Geranic Acid and Nerolic Acid)

According to the approaches in the literature [23], a solution of NaClO_2_ (22.0 g, 30 mmol) and NaH_2_PO_4_·2H_2_O (34.4 g, 22 mmol) in water was added dropwise to a solution of (*E*)-3,7-dimethyl-2,6-octadienal (4.0 g, 22 mmol) and 2-methyl-2-butene in acetone (220 mL) at room temperature and stirred for 4 h, the solution was extracted with ethyl acetate, and the organic layer was washed with brine, dried over anhydrous Na_2_SO_4_. The solvent was removed under reduced pressure and the residue was purified by column chromatography using silica gel (petroleum ether/EtOAc/CH_3_COOH 25:1:0.5) to afford (*E*)-3,7-dimethyl-2,6-octadienoic acid (4.0 g, 90%) as a colorless oil; the (*Z*)-isomer (8.5 g, 86%) was also prepared as a colorless oil using (*Z*)-3,7-dimethyl- 2,6-octadienal (12.3 g) as the starting material following the same procedure [28].

#### 3.2.3. General Procedure for the Synthesis of Compounds **5**

Take compound **5a** as an example: according to the procedure in the literature [22], SOCl_2_ (1.4 mL) was added to a solution of (*E*)-3,7-dimethyl-2,6-octadienoic acid (0.8 g, 5 mmol) in 40 mL DCM in a 100 mL flask at room temperature. The mixture was stirred and heated at 40 °C for 4 h. Then cool down to room temperature and remove the solvent under reduced pressure. The residue was dissolved in 10 mL DCM, the 10 mL DCM solution of aniline (0.92 mL, 10 mmol) was added and stirred for 10 h at room temperature. Quenched the reaction with water, extracted with DCM, and the organic layer was washed with brine, dried over anhydrous Na_2_SO_4_. Removed the solvent under reduced pressure and the residue was purified by column chromatography to afford compound **5a**.

*(E)-3,7-Dimethyl-N-phenyl-2,6-octadienamide* (**5a**), grey oil, yield 79%. ^1^H-NMR (300 MHz, CDCl_3_) δ: 7.53 (d, *J* = 7.9 Hz, 2H, ArH), 7.34–7.29 (m, 2H, ArH), 7.11–7.06 (m, 2H, ArH + NH), 5.69 (s, 1H, =CH), 5.14–5.05 (m, 1H, =CH), 2.22 (d, *J* = 1.2 Hz, 3H, CH_3_), 2.17 (br.s, 4H, 2 × CH_2_), 1.70 (s, 3H, CH_3_), 1.62 (s, 3H, CH_3_); HR-MS (ESI) *m*/*z*: C_16_H_21_NO [M + H]^+^, Calcd. 244.1696; Found 244.1693. The spectral data were identical with those in the reference [29].

*(E)-N-(2,4-Dichlorophenyl)-3,7-dimethyl-2,6-octadienamide* (**5b**), pale yellow oil, yield 78%. ^1^H-NMR (300 MHz, CDCl_3_) δ: 8.44 (d, *J* = 8.9 Hz, 1H, ArH), 7.50 (s, 1H, NH), 7.37 (d, *J* = 2.4 Hz, 1H, ArH), 7.24 (dd, *J* = 8.9, 2.4 Hz, 1H, ArH), 5.73 (s, 1H, =CH), 5.13–5.04 (m, 1H, =CH), 2.23 (d, *J* = 1.2 Hz, 3H, CH_3_), 2.20–2.18 (m, 4H, 2 × CH_2_), 1.70 (s, 3H, CH_3_), 1.63 (s, 3H, CH_3_); HR-MS (ESI) *m*/*z*: C_16_H_19_Cl_2_NO [M + H]^+^, Calcd. 312.0916; Found 312.0917.

*(E)-N-(2-Chlorophenyl)-3,7-dimethyl-2,6-octadienamide* (**5c**), yellow oil, yield 69%. ^1^H-NMR (300 MHz, CDCl_3_) δ: 8.45 (d, *J* = 8.1 Hz, 1H, ArH), 7.57 (s, 1H, NH), 7.35 (dd, *J* = 8.1, 1.5 Hz, 1H, ArH), 7.30–7.23 (m, 1H, ArH), 7.04–6.98 (m, 1H, ArH), 5.75 (s, 1H, =CH), 5.14–5.04 (m, 1H, =CH), 2.23 (d, *J* = 1.2 Hz, 3H, CH_3_), 2.20 (br.s, 4H, 2 × CH_2_), 1.71 (s, 3H, CH_3_), 1.64 (s, 3H, CH_3_); HR-MS (ESI) *m*/*z*: C_16_H_20_ClNO [M + H]^+^, Calcd. 278.1306; Found 278.1303.

*(E)-N-(2-Fluorophenyl)-3,7-dimethyl-2,6-octadienamide* (**5d**), brown oil, yield 35%. ^1^H-NMR (300 MHz, CDCl_3_) δ: 8.42–8.36 (m, 1H, ArH), 7.29 (s, 1H, NH), 7.15–6.99 (m, 3H, ArH), 5.73 (s, 1H, =CH), 5.16–5.09 (m, 1H, =CH), 2.23 (d, *J* = 1.2 Hz, 3H, CH_3_), 2.19 (br.s, 4H, 2 × CH_2_),1.70 (s, 3H, CH_3_), 1.63 (s, 3H, CH_3_); HR-MS (ESI) *m*/*z*: C_16_H_20_FNO [M + H]^+^, Calcd. 262.1601; Found 262.1600.

*(E)-N-(4-Trifluoromethylphenyl)-3,7-dimethyl-2,6-octadienamide* (**5e**), yellow oil, yield 16%. ^1^H-NMR (300 MHz, CDCl_3_) δ: 7.66 (d, *J* = 8.7 Hz, 2H, ArH), 7.55 (d, *J* = 8.7 Hz, 2H, ArH), 7.26 (s, 1H, NH), 5.70 (s, 1H, =CH), 5.15–5.08 (m, 1H, =CH), 2.23 (d, *J* = 1.2 Hz, 3H, CH_3_), 2.19 (br.s, 4H, 2 × CH_2_), 1.70 (s, 3H, CH_3_), 1.62 (s, 3H, CH_3_); HR-MS (ESI) *m*/*z*: C_17_H_20_F_3_NO [M + H]^+^, Calcd. 312.1569; Found 312.1568.

*(E)-N-(4-Methylphenyl)-3,7-dimethyl-2,6-octadienamide* (**5f**), brown oil, yield 74%. ^1^H-NMR (300 MHz, CDCl_3_) δ: 7.42 (d, *J* = 7.5 Hz, 2H, ArH), 7.11 (d, *J* = 7.5 Hz, 3H, ArH + NH), 5.68 (s, 1H, =CH), 5.14–5.07 (m, 1H, =CH), 2.31 (s, 3H, ArCH_3_), 2.21 (d, *J* = 1.2 Hz, 3H, CH_3_), 2.18–2.15 (m, 4H, 2 × CH_2_), 1.70 (s, 3H, CH_3_), 1.62 (s, 3H, CH_3_); HR-MS (ESI) *m*/*z*: C_17_H_23_NO [M + H]^+^, Calcd. 258.1852; Found 258.1852.

*(E)-N-Phenyl-N,3,7-trimethyl-2,6-octadienamide* (**5g**), brown oil, yield 76%. ^1^H-NMR (300 MHz, CDCl_3_) δ: 7.40–7.34 (m, 2H, ArH), 7.30–7.24 (m, 1H, ArH), 7.18–7.14 (m, 2H, ArH), 5.47 (br.s, 1H, =CH), 4.96–4.91 (m, 1H, =CH), 3.32 (s, 3H, NCH_3_), 2.10 (d, *J* = 1.2 Hz, 3H, CH_3_), 1.95 (br.s, 4H, 2 × CH_2_), 1.63 (s, 3H, CH_3_), 1.51 (s, 3H, CH_3_); HR-MS (ESI) *m*/*z*: C_17_H_23_NO [M + H]^+^, Calcd. 258.1852; Found 258.1850. The spectral data were identical with those in the reference [30].

*(E)-N-Benzyl-3,7-dimethyl-2,6-octadienamide* (**5h**), yellow oil, yield 71%. ^1^H-NMR (300 MHz, CDCl_3_) δ: 7.36–7.25 (m, 5H, ArH), 5.66 (br.s, 1H, NH), 5.56 (s, 1H, =CH), 5.10–5.05 (m, 1H, =CH), 4.47 (d, *J* = 6.0 Hz, 2H, ArCH_2_), 2.18 (d, *J* = 1.2 Hz, 3H, CH_3_), 2.14–2.08 (m, 4H, 2 × CH_2_), 1.68 (s, 3H, CH_3_), 1.60 (s, 3H, CH_3_); HR-MS (ESI) *m*/*z*: C_17_H_23_NO [M + H]^+^, Calcd. 258.1852; Found 258.1850. The spectral data were identical with those in the reference [31].

*(E)-N-(4-Fluorobenzyl)-3,7-dimethyl-2,6-octadienamide* (**5i**), yellow oil, yield 73%. ^1^H-NMR (300 MHz, CDCl_3_) δ: 7.30–7.23 (m, 2H, ArH), 7.05–6.98 (m, 2H, ArH), 5.64 (br.s, 1H, NH), 5.55 (s, 1H, =CH), 5.09–5.04 (m, 1H, =CH), 4.44 (d, *J* = 5.8 Hz, 2H, ArCH_2_), 2.18 (d, *J* = 1.2 Hz, 3H, CH_3_), 2.14–2.08 (m, 4H, 2 × CH_2_), 1.68 (s, 3H, CH_3_), 1.60 (s, 3H, CH_3_); HR-MS (ESI) *m*/*z*: C_17_H_22_FNO [M + H]^+^: Calcd. 276.1758; Found 276.1754.

*(E)-N-(4-Methoxybenzyl)-3,7-dimethyl-2,6-octadienamide* (**5j**), yellow oil, yield 53%. **^1^**H-NMR (300 MHz, CDCl_3_) δ: 7.23 (d, *J* = 8.7 Hz, 2H, ArH), 6.86 (d, *J* = 8.7 Hz, 2H, ArH), 5.57 (s, 1H, NH), 5.53 (s, 1H, =CH), 5.08–5.05 (m, 1H, =CH), 4.41 (d, *J* = 6.0 Hz, 2H, ArCH_2_), 3.80 (s, 3H, OCH_3_), 2.17 (d, *J* = 1.2 Hz, 3H, CH_3_), 2.13–2.08 (m, 4H, 2 × CH_2_), 1.68 (s, 3H, CH_3_), 1.60 (s, 3H, CH_3_); HR-MS (ESI) *m*/*z*: C_18_H_25_NO_2_ [M + H]^+^: Calcd. 288.1958; Found 288.1954.

*(E)-N-(2-Chlorobenzyl)-3,7-dimethyl-2,6-octadienamide* (**5k**), yellow oil, 45%. ^1^H-NMR (300 MHz, CDCl_3_) δ: 7.43–7.32 (m, 2H, ArH), 7.24–7.18 (m, 2H, ArH), 5.83 (s, 1H, NH), 5.57 (s, 1H, =CH), 5.08–5.04 (m, 1H, =CH), 4.55 (d, *J* = 6.0 Hz, 2H, CH_2_), 2.15 (d, *J* = 1.2 Hz, 3H, CH_3_), 2.11 (br.s, 4H, 2 × CH_2_), 1.67 (s, 3H, CH_3_), 1.59 (s, 3H, CH_3_); HR-MS (ESI) *m*/*z*: C_17_H_22_ClNO [M + H]^+^: Calcd. 292.1462; Found 292.1460.

*(E)-3,7-Dimethyl-1-morpholino-2,6-octadien-1-one* (**5l**), yellow oil, yield 70%. ^1^H-NMR (300 MHz, CDCl_3_) δ: 5.72 (s, 1H, =CH), 5.09–5.05 (m, 1H, =CH), 3.67 (br, 6H, 3 × CH_2_), 3.50 (br, 2H, CH_2_), 2.17–2.13 (m, 4H, 2 × CH_2_), 1.88 (d, *J* = 1.2 Hz, 3H, CH_3_), 1.67 (s, 3H, CH_3_), 1.61 (s, 3H, CH_3_); HR-MS (ESI) *m*/*z*: C_14_H_23_NO_2_ [M + H]^+^, Calcd. 238.1800; Found 238.1801.

*(E)-3,7-Dimethyl-1-(pyrrolidin-1-yl)-2,6-octadien-1-one* (**5m**), colorless oil, yield 61%. ^1^H-NMR (300 MHz, CDCl_3_) δ: 5.77 (s, 1H, =CH), 5.10–5.07 (m, 1H, =CH), 3.50 (t, *J* = 6.6 Hz, 2H, NCH_2_), 3.42 (t, *J* = 6.6 Hz, 2H, NCH_2_), 2.18–2.12 (m, 4H, 2 × CH_2_), 2.05 (d, *J* = 1.2 Hz, 3H, CH_3_), 1.95–1.83 (m, 4H, 2 × CH_2_), 1.69 (s, 3H, CH_3_), 1.61 (s, 3H, CH_3_); HR-MS (ESI) *m*/*z*: C_14_H_23_NO [M + H]^+^, Calcd. 222.1852; Found 222.1849. The spectral data were identical with those in the reference [28].

*(E)-N-Isopropyl-3,7-dimethyl-2,6-octadienamide* (**5n**), yellow oil, yield 70%. ^1^H-NMR (300 MHz, CDCl_3_) δ: 5.51 (s, 1H, =CH), 5.27 (br.s, 1H, NH), 5.11–5.05 (m, 1H, =CH), 4.15–4.08 (m, 1H, NCH), 2.14 (d, *J* = 1.2 Hz, 3H, CH_3_), 2.12–2.06 (m, 4H, 2 × CH_2_), 1.68 (s, 3H, CH_3_), 1.61 (s, 3H, CH_3_), 1.16 (d, *J* = 6.6 Hz, 6H, 2 × CH_3_); HR-MS (ESI) *m*/*z*: C_12_H_21_NO [M + H]^+^, Calcd. 196.1696; Found 196.1694. The spectral data were identical with those in the reference [29].

*(Z)-N-Phenyl-3,7-dimethyl-2,6-octadienamide* (**5A**), brown oil, yield 70%. ^1^H-NMR (300 MHz, CDCl_3_) δ: 7.52 (d, *J* = 7.8 Hz, 2H, ArH), 7.33–7.28 (m, 2H, ArH), 7.16 (s, 1H, NH), 7.11–7.06 (m, 1H, ArH), 5.70 (s, 1H, =CH), 5.22–5.16 (m, 1H, =CH), 2.70 (t, *J* = 7.8 Hz, 2H, CH_2_), 2.26–2.18 (m, 2H, CH_2_), 1.89 (d, *J* = 1.2 Hz, 3H, CH_3_), 1.68 (s, 3H, CH_3_), 1.63 (s, 3H, CH_3_); HR-MS (ESI) *m*/*z*: C_16_H_21_NO [M + H]^+^, Calcd. 244.1696; Found 244.1691. The spectral data were identical with those in the reference [29].

*(Z)-N-(2,4-Dichlorophenyl)-3,7-dimethyl-2,6-octadienamide* (**5B**), yellow oil, yield 65%. ^1^H-NMR (300 MHz, CDCl_3_) δ: 8.43 (d, *J* = 8.7 Hz, 1H, ArH), 7.47 (s, 1H, NH), 7.37 (d, *J* = 2.4 Hz, 1H, ArH), 7.22 (dd, *J* = 8.7, 2.4 Hz, 1H, ArH), 5.73 (s, 1H, =CH), 5.18–5.14 (m, 1H, =CH), 2.71 (t, *J* = 7.5 Hz, 2H, CH_2_), 2.26–2.17 (m, 2H, CH_2_), 1.93 (d, *J* = 1.2 Hz, 3H, CH_3_), 1.67 (s, 3H, CH_3_), 1.63 (s, 3H, CH_3_); HR-MS (ESI) *m*/*z*: C_16_H_19_Cl_2_NO [M + H]^+^, Calcd. 312.0916; Found 312.0915.

*(Z)-N-(2-Chlorophenyl)-3,7-dimethyl-2,6-octadienamide* (**5C**), brown oil, yield 67%. ^1^H-NMR (300 MHz, CDCl_3_) δ: 8.44 (d, *J* = 8.0 Hz, 1H, ArH), 7.54 (s, 1H, NH), 7.34 (dd, *J* = 8.0, 1.5 Hz, 1H, ArH), 7.28–7.23 (m, 1H, ArH), 7.04–6.98 (m, 1H, ArH), 5.75 (s, 1H, =CH), 5.19–5.14 (m, 1H, CH), 2.72 (t, *J* = 7.5 Hz, 2H, CH_2_), 2.24–2.18 (m, 2H, CH_2_), 1.93 (d, *J* = 1.2 Hz, 3H, CH_3_), 1.68 (s, 3H, CH_3_), 1.63 (s, 3H, CH_3_); HR-MS (ESI) *m*/*z*: C_16_H_20_ClNO [M + H]^+^, Calcd. 278.1306; Found 278.1304.

*(Z)-N-(2-Fluorophenyl)-3,7-dimethyl-2,6-octadienamide* (**5D**), brown oil, 15%. ^1^H-NMR (300 MHz, CDCl_3_) δ: 8.43–8.36 (m, 1H, ArH), 7.26 (s, 1H, NH), 7.15–6.99 (m, 3H, ArH), 5.73 (s, 1H, =CH), 5.19–5.10 (m, 1H, =CH), 2.72 (t, *J* = 7.5 Hz, 2H, CH_2_), 2.24–2.17 (m, 2H, CH_2_), 1.92 (d, *J* = 1.2 Hz, 3H, CH_3_), 1.70 (s, 3H, CH_3_), 1.63 (s, 3H, CH_3_); HR-MS (ESI) *m*/*z*: C_16_H_20_FNO [M + H]^+^, Calcd. 262.1601; Found 262.1597.

*(Z)-3,7-Dimethyl-N-(4-(trifluoromethyl)phenyl)octa-2,6-dienamide* (**5E**), brown oil, yield 16%. ^1^H-NMR (300 MHz, CDCl_3_) δ: 7.66 (d, *J* = 8.7 Hz, 2H, ArH), 7.55 (d, *J* = 8.7 Hz, 2H, ArH), 7.29 (s, 1H, NH), 5.71 (s, 1H, =CH), 5.19–5.11 (m, 1H, =CH), 2.71 (t, *J* = 7.5 Hz, 2H, CH_2_), 2.26–2.21 (m, 2H, CH_2_), 1.92 (d, *J* = 1.2 Hz, 3H, CH_3_), 1.69 (s, 3H, CH_3_), 1.63 (s, 3H, CH_3_); HR-MS (ESI) *m*/*z*: C_17_H_20_F_3_NO [M + H]^+^, Calcd. 312.1569; Found 312.1565.

*(Z)-N-(4-Methylphenyl)-3,7-dimethyl-2,6-octadienamide* (**5F**), brown oil, yield 60%. ^1^H-NMR (300 MHz, CDCl_3_) δ: 7.40 (d, *J* = 7.8 Hz, 2H, ArH), 7.11 (d, *J* = 7.8 Hz, 2H, ArH), 7.07 (s, 1H, NH), 5.68 (s, 1H, =CH), 5.20–5.16 (m, 1H, =CH), 2.69 (t, *J* = 7.5 Hz, 2H, CH_2_), 2.31 (s, 3H, CH_3_), 2.25–2.16 (m, 2H, CH_2_), 1.89 (d, *J* = 1.2 Hz, 3H, CH_3_), 1.68 (s, 3H, CH_3_), 1.62 (s, 3H, CH_3_); HR-MS (ESI) *m*/*z*: C_17_H_23_NO [M + H]^+^, Calcd. 258.1852; Found 258.1850.

*(Z)-N-Phenyl-N,3,7-trimethyl-2,6-octadienamide* (**5G**), brown oil, yield 67%. ^1^H-NMR (300 MHz, CDCl_3_) δ: 7.40–7.34 (m, 2H, ArH), 7.30–7.24 (m, 1H, ArH), 7.18–7.14 (m, 2H, ArH), 5.46 (br.s, 1H, =CH), 5.22–5.16 (m, 1H, =CH), 3.31 (s, 3H, NCH_3_), 2.61 (t, *J* = 7.5 Hz, 2H, CH_2_), 2.22–2.14 (m, 2H, CH_2_), 1.70 (d, *J* = 1.2 Hz, 3H, CH_3_), 1.67 (s, 3H, CH_3_), 1.64 (s, 3H, CH_3_); HR-MS (ESI) *m*/*z*: C_17_H_23_NO [M + H]^+^, Calcd. 258.1852; Found 258.1849. The spectral data were identical with those in the reference [31].

*(Z)-N-Benzyl-3,7-dimethyl-2,6-octadienamide* (**5H**), yellow oil, yield 57%. ^1^H-NMR (300 MHz, CDCl_3_) δ: 7.31–7.26 (m, 5H, ArH), 5.66 (br.s, 1H, NH), 5.58 (s, 1H, =CH), 5.18–5.12 (m, 1H, =CH), 4.47 (d, *J* = 6.0 Hz, 2H, ArCH_2_), 2.64 (t, *J* = 7.8 Hz, 2H, CH_2_), 2.22–2.18 (m, 2H, CH_2_), 1.84 (d, *J* = 1.2 Hz, 3H, CH_3_), 1.64 (s, 3H, CH_3_), 1.60 (s, 3H, CH_3_); HR-MS (ESI) *m*/*z*: C_17_H_23_NO [M + H]^+^, Calcd. 258.1852; Found 258.1848. The spectral data were identical with those in the reference [30].

*(Z)-N-(4-Fluorobenzyl)-3,7-dimethyl-2,6-octadienamide* (**5I**), brown oil, yield 72%. ^1^H-NMR (300 MHz, CDCl_3_) δ: 7.29–7.23 (m, 2H, ArH), 7.03–6.97 (m, 2H, ArH), 5.71 (br.s, 1H, NH), 5.58 (s, 1H, =CH), 5.17–5.11 (m, 1H, =CH), 4.43 (d, *J* = 5.8 Hz, 2H, ArCH_2_), 2.64 (t, *J* = 7.2 Hz, 2H, CH_2_), 2.21–2.16 (m, 2H, CH_2_), 1.84 (d, *J* = 1.2 Hz, 3H, CH_3_), 1.65 (s, 3H, CH_3_), 1.60 (s, 3H, CH_3_); HR-MS (ESI) *m*/*z*: C_17_H_22_FNO [M + H]^+^: Calcd. 276.1758; Found 276.1754.

*(Z)-N-(4-Methoxybenzyl)-3,7-dimethyl-2,6-octadienamide* (**5J**), yellow oil, 27%. ^1^H-NMR (300 MHz, CDCl_3_) δ: 7.20 (d, *J* = 8.4 Hz, 2H, ArH), 6.84 (d, *J* = 8.4 Hz, 2H, ArH), 5.71 (s, 1H, NH), 5.56 (s, 1H, =CH), 5.17–5.11 (m, 1H, =CH), 4.38 (d, *J* = 6.0 Hz, 2H, ArCH_2_), 3.79 (s, 3H, OCH_3_), 2.64 (t, *J* = 7.5 Hz, 2H, CH_2_), 2.21–2.13 (m, 2H, CH_2_), 1.83 (d, *J* = 1.2 Hz, 3H, CH_3_), 1.64 (s, 3H, CH_3_), 1.60 (s, 3H, CH_3_); HR-MS (ESI) *m*/*z*: C_18_H_25_FNO_2_ [M + H]^+^: Calcd. 288.1958; Found 288.1955.

*(Z)-N-(2-Chlorobenzyl)-3,7-dimethyl-2,6-octadienamide* (**5K**), yellow oil, 40%. ^1^H-NMR (300 MHz, CDCl_3_) δ: 7.40–7.33 (m, 2H, ArH), 7.24–7.19 (m, 2H, ArH), 5.90 (s, 1H, NH), 5.59 (s, 1H, =CH), 5.15–5.10 (m, 1H, =CH), 4.54 (d, *J* = 6.0 Hz, 2H, CH_2_), 2.63 (t, *J* = 7.5 Hz, 2H, CH_2_), 2.20–2.11 (m, 2H, CH_2_), 1.83 (d, *J* = 1.2 Hz, 3H, CH_3_), 1.64 (s, 3H, CH_3_), 1.59 (s, 3H, CH_3_); HR-MS (ESI) *m*/*z*: C_17_H_22_ClNO [M + H]^+^: Calcd. 292.1462; Found 292.1461.

*(Z)-3,7-Dimethyl-1-morpholino-2,6-octadien-1-one* (**5L**), yellow oil, yield 60%. ^1^H-NMR (300 MHz, CDCl_3_) δ: 5.74 (s, 1H, =CH), 5.13–5.07 (m, 1H, =CH), 3.66 (br, 6H, 3 × CH_2_), 3.50 (br, 2H, CH_2_), 2.33 (t, *J* = 7.5 Hz, 2H, CH_2_), 2.18–2.10 (m, 2H, CH_2_), 1.83 (d, *J* = 1.2 Hz, 3H, CH_3_), 1.68 (s, 3H, CH_3_), 1.61 (s, 3H, CH_3_); HR-MS (ESI) *m*/*z*: C_14_H_23_NO_2_ [M + H]^+^, Calcd. 238.1802; Found 238.1800.

*(Z)-3,7-Dimethyl-1-(pyrrolidin-1-yl)-2,6-octadien-1-one* (**5M**), pale yellow oil, yield 65%. ^1^H-NMR (300 MHz, CDCl_3_) δ: 5.77 (s, 1H, =CH), 5.18–5.11 (m, 1H, =CH), 3.49 (t, *J* = 6.9 Hz, 2H, NCH_2_), 3.42 (t, *J* = 6.9 Hz, 2H, NCH_2_), 2.53 (t, *J* = 7.5 Hz, 2H, CH_2_), 2.20–2.15 (m, 2H, CH_2_), 1.95–1.82 (m, 7H, 2 × CH_2_ + CH_3_), 1.67 (s, 3H, CH_3_), 1.61 (s, 3H, CH_3_); HR-MS (ESI) *m*/*z*: C_14_H_23_NO [M + H]^+^, Calcd. 222.1852; Found 222.1850. The spectral data were identical with those in the reference [28].

*(Z)-N-Isopropyl-3,7-dimethyl-2,6-octadienamide* (**5N**), yellow oil, yield 62%. ^1^H-NMR (300 MHz, CDCl_3_) δ: 5.51 (s, 1H, =CH), 5.27 (br.s, 1H, NH), 5.19–5.14 (m, 1H, =CH), 4.15–4.08 (m, 1H, NCH), 2.59 (t, *J* = 7.2 Hz, 2H, CH_2_), 2.21–2.14 (m, 2H, CH_2_), 1.86 (d, *J* = 1.2 Hz, 3H, CH_3_), 1.69 (s, 3H, CH_3_), 1.62 (s, 3H, CH_3_), 1.16 (d, *J* = 6.6 Hz, 6H, 2 × CH_3_); HR-MS (ESI) *m*/*z*: C_12_H_21_NO [M + H]^+^, Calcd. 196.1696; Found 196.1694. The spectral data were identical with those in the reference [28].

#### 3.2.4. General Procedure for the Synthesis of Compounds **6**

Take compound **6a** as an example: according to the approach in the literature [25,32], compound **5a** 0.24 g (1 mmol), CH_3_COOOH (2 mL), Na_2_CO_3_ (0.7 g) and DCM (10 mL) were added to a 50 mL flask and stirred at room temperature for 2–4 h, quenched the reaction with water, and extracted with DCM. The organic layer was washed with brine and dried over anhydrous Na_2_SO_4_. The solvent was removed under reduce pressure, and the residue was purified by column chromatography to give the compounds **6a**.

*(E)-N-Phenyl-5-(3,3-dimethyloxiran-2-yl)-3-methylpent-2-enamide* (**6a**), yellow solid, yield 80%. m.p. 82–84 °C, ^1^H-NMR (300 MHz, CDCl_3_) δ: 7.54 (d, *J* = 7.8 Hz, 2H, ArH), 7.29 (t, *J* = 7.8 Hz, 2H, ArH), 7.21 (s, 1H, NH), 7.09 (t, *J* = 7.8 Hz, 1H, ArH), 5.76 (s, 1H, =CH), 2.74 (dd, J = 5.1, 7.2Hz, 1H, OCH), 2.36–2.25 (m, 2H, CH_2_), 2.24 (d, *J* = 1.2 Hz, 3H, CH_3_), 1.78–1.66 (m, 2H, CH_2_), 1.33 (s, 3H, CH_3_), 1.29 (s, 3H, CH_3_); HR-MS (ESI) *m*/*z*: C_16_H_21_NO_2_ [M + H]^+^, Calcd. 260.1645; Found 260.1643.

*(E)-N-(2,4-Dichlorophenyl)-5-(3,3-dimethyloxiran-2-yl)-3-methylpent-2-enamide* (**6b**), yellow oil, yield 63%. ^1^H-NMR (300 MHz, CDCl_3_) δ: 8.42 (d, *J* = 8.7 Hz, 1H, ArH), 7.53 (s, 1H, NH), 7.26 (d, *J* = 2.4 Hz, 1H, ArH), 7.24 (dd, *J* = 8.7, 2.4 Hz, 1H, ArH), 5.80 (s, 1H, =CH), 2.74 (dd, *J* = 7.2, 5.1 Hz, 1H, OCH), 2.40–2.30 (m, 2H, CH_2_), 2.25 (d, *J* = 1.2 Hz, 3H, CH_3_), 1.82–1.66 (m, 2H, CH_2_), 1.33 (s, 3H, CH_3_), 1.30 (s, 3H, CH_3_); HR-MS (ESI) *m*/*z*: C_16_H_19_Cl_2_NO_2_ [M + H]^+^, Calcd. 328.0866; Found 328.0864.

*(E)-N-(2-Chlorophenyl)-5-(3,3-dimethyloxiran-2-yl)-3-methylpent-2-enamide* (**6c**), yellow oil, yield 80%. ^1^H-NMR (300 MHz, CDCl_3_) δ: 8.44 (d, *J* = 7.8 Hz, 1H, ArH), 7.59 (s, 1H, NH), 7.36 (dd, *J* = 7.8, 1.8 Hz, 1H, ArH), 7.30–7.23 (m, 1H, ArH), 7.05–6.99 (m, 1H, ArH), 5.81 (s, 1H, =CH), 2.74 (dd, *J* = 7.2, 5.4 Hz, 1H, OCH), 2.40–2.29 (m, 2H, CH_2_), 2.25 (d, *J* = 1.2 Hz, 3H, CH_3_), 1.82–1.67 (m, 2H, CH_2_), 1.33 (s, 3H, CH_3_), 1.30 (s, 3H, CH_3_); HR-MS (ESI) *m*/*z*: C_16_H_20_ClNO_2_ [M + H]^+^, Calcd. 294.1255; Found 294.1257.

*(E)-N-(4-Methylphenyl)-5-(3,3-dimethyloxiran-2-yl)-3-methylpent-2-enamide* (**6d**), colorless oil, yield 75%. ^1^H-NMR (300 MHz, CDCl_3_) δ: 7.42 (d, *J* = 7.8 Hz, 2H, ArH), 7.11 (d, *J* = 7.8 Hz,, 3H, ArH + NH), 5.74 (s, 1H, =CH), 2.74 (dd, *J* = 7.2, 5.1 Hz, 1H, OCH), 2.35–2.25 (m, 5H, CH_2_ + CH_3_), 2.23 (d, *J* = 1.2 Hz, 3H, CH_3_), 1.80–1.64 (m, 2H, CH_2_), 1.32 (s, 3H, CH_3_), 1.29 (s, 3H, CH_3_); HR-MS (ESI) *m*/*z*: C_17_H_23_NO_2_ [M + H]^+^, Calcd. 274.1802; Found 274.1798.

*(E)-N-Phenyl-5-(3,3-dimethyloxiran-2-yl)-N,3-dimethylpent-2-enamide* (**6e**), yellow oil, yield 96%. ^1^H-NMR (300 MHz, CDCl_3_) δ: 7.40–7.33 (m, 2H, ArH), 7.31–7.25 (m, 1H, ArH), 7.17–7.13 (m, 2H, ArH), 5.51 (br.s, 1H, =CH), 3.32 (s, 3H, CH_3_), 2.55 (t, *J* = 6.3 Hz, 1H, OCH), 2.11 (d, *J* = 1.2 Hz, 3H, CH_3_), 2.08–2.03 (m, 2H, CH_2_),1.53–1.45 (m, 2H, CH_2_), 1.25 (s, 3H, CH_3_), 1.18 (s, 3H, CH_3_); HR-MS (ESI) *m*/*z*: C_17_H_23_NO_2_ [M + H]^+^, Calcd. 274.1802; Found 274.1792.

*(E)-N-Benzyl-5-(3,3-dimethyloxiran-2-yl)-3-methylpent-2-enamide* (**6f**), yellow oil, yield 80%. ^1^H-NMR (300 MHz, CDCl_3_) δ: 7.36–7.25 (m, 5H, ArH), 5.68 (br.s, 1H, NH), 5.62 (s, 1H, =CH), 4.47 (d, *J* = 6.0 Hz, 2H, ArCH_2_), 2.71 (dd, *J* = 7.2, 5.4 Hz, 1H, OCH), 2.30–2.22 (m, 2H, CH_2_), 2.20 (d, *J* = 1.2 Hz, 3H, CH_3_), 1.75–1.62 (m, 2H, CH_2_), 1.32 (s, 3H, CH_3_), 1.26 (s, 3H, CH_3_); HR-MS (ESI) *m*/*z*: C_17_H_23_NO_2_ [M + H]^+^, Calcd. 274.1802; Found 274.1799.

*(E)-N-(4-Fluorobenzyl)-5-(3,3-dimethyloxiran-2-yl)-3-methylpent-2-enamide* (**6g**), white solid, yield 84%. m.p. 50–52 °C, ^1^H-NMR (300 MHz, CDCl_3_) δ: 7.29–7.23 (m, 2H, ArH), 7.04–6.97 (m, 2H, ArH), 5.76 (br.s, 1H, NH), 5.62 (s, 1H, =CH), 4.43 (d, *J* = 5.8 Hz, 2H, ArCH_2_), 2.71 (dd, *J* = 7.2, 5.1 Hz, 1H, OCH), 2.30–2.19 (m, 5H, CH_2_ + CH_3_), 1.76–1.62 (m, 2H, CH_2_), 1.30 (s, 3H, CH_3_), 1.26 (s, 3H, CH_3_); HR-MS (ESI) *m*/*z*: C_17_H_22_FNO_2_ [M + H]^+^, Calcd. 292.1707; Found 292.1706.

*(E)-5-(3,3-Dimethyloxiran-2-yl)-3-methyl-1-morpholinopent-2-en-1-one* (**6h**), pale yellow oil, yield 71%. ^1^H-NMR (300 MHz, CDCl_3_) δ: 5.81 (s, 1H, =CH), 3.67 (br, 6H, 3 × CH_2_), 3.51 (br, 2H, CH_2_), 2.71 (dd, *J* = 7.2, 5.1 Hz, 1H, OCH), 2.32–2.23 (m, 2H, CH_2_), 1.92 (d, *J* = 1.2 Hz, 3H, CH_3_), 1.81–1.63 (m, 2H, CH_2_), 1.31 (s, 3H, CH_3_), 1.25 (s, 3H, CH_3_); HR-MS (ESI) *m*/*z*: C_14_H_23_NO_3_ [M + H]^+^, Calcd. 254.1751; Found 254.1747.

*(E)-5-(3,3-Dimethyloxiran-2-yl)-3-methyl-1-(pyrrolidin-1-yl)pent-2-en-1-one* (**6i**), yellow oil, yield 70%. ^1^H-NMR (300 MHz, CDCl_3_) δ: 5.84 (s, 1H, =CH), 3.50 (t, *J* = 6.6 Hz, 2H, NCH_2_), 3.43 (t, *J* = 6.6 Hz, 2H, NCH_2_), 2.72 (dd, *J* = 6.9, 5.7 Hz, 1H, OCH), 2.32–2.22 (m, 2H, CH_2_), 2.09 (d, *J* = 1.2 Hz, 3H, CH_3_), 1.96–1.65 (m, 6H, 3 × CH_2_), 1.31 (s, 3H, CH_3_), 1.28 (s, 3H, CH_3_); HR-MS (ESI) *m*/*z*: C_14_H_23_NO_2_ [M + H]^+^, Calcd. 238.1802; Found 238.1799.

*(E)-N-Isopropyl-5-(3,3-dimethyloxiran-2-yl)-3-methylpent-2-enamide* (**6j**), yellow oil, yield 79%. ^1^H-NMR (300 MHz, CDCl_3_) δ: 5.55 (s, 1H, =CH), 5.24 (br.s, 1H, NH), 4.16–4.08 (m, 1H, NCH), 2.72 (dd, *J* = 7.0, 5.4 Hz, 1H, OCH), 2.29–2.14 (m, 5H, CH_2_ + CH_3_), 1.76–1.62 (m, 2H, CH_2_), 1.31 (s, 3H, CH_3_), 1.27 (s, 3H, CH_3_), 1.16 (d, *J* = 6.5 Hz, 6H, 2 × CH_3_); HR-MS (ESI) *m*/*z*: C_13_H_24_NO_2_ [M + H]^+^, Calcd. 226.1807; Found 226.1799.

*(Z)-N-Phenyl-5-(3,3-dimethyloxiran-2-yl)-3-methylpent-2-enamide* (**6A**), brown oil, yield 80%. ^1^H-NMR (300 MHz, CDCl_3_) δ: 7.62 (s, 1H, NH), 7.54 (d, *J* = 7.5 Hz, 2H, ArH), 7.33–7.26 (m, 2H, ArH), 7.10–7.04 (m, 1H, ArH), 5.76 (s, 1H, =CH), 2.95–2.60 (m, 3H, OCH + CH_2_), 1.89 (d, *J* = 1.2 Hz, 3H, CH_3_), 1.82–1.72 (m, 2H, CH_2_), 1.30 (s, 3H, CH_3_), 1.29 (s, 3H, CH_3_); HR-MS (ESI) *m*/*z*: C_16_H_21_NO_2_ [M + H]^+^, Calcd. 260.1645; Found 260.1641.

*(Z)-N-(2,4-Dichlorophenyl)-5-(3,3-dimethyloxiran-2-yl)-3-methylpent-2-enamide (***6B**), pale yellow oil, yield 68%. ^1^H-NMR (300 MHz, CDCl_3_) δ: 8.37 (d, *J* = 8.7 Hz, 1H, ArH), 7.57 (s, 1H, NH), 7.35 (d, *J* = 2.1 Hz, 1H, ArH), 7.21 (dd, *J* = 8.7, 2.1 Hz, 1H, ArH), 5.79 (s, 1H, =CH), 2.98–2.73 (m, 3H, OCH + CH_2_), 1.93 (d, *J* = 1.2 Hz, 3H, CH_3_), 1.82–1.74 (m, 2H, CH_2_), 1.30 (s, 3H, CH_3_), 1.29 (s, 3H, CH_3_); HR-MS (ESI) *m*/*z*: C_16_H_19_Cl_2_NO_2_ [M + H]^+^, Calcd. 328.0866; Found 328.0865.

*(Z)-N-(2-Chlorophenyl)-5-(3,3-dimethyloxiran-2-yl)-3-methylpent-2-enamide* (**6C**), yellow oil, yield 76%. ^1^H-NMR (300 MHz, CDCl_3_) δ: 8.42 (d, *J* = 8.4 Hz, 1H, ArH), 7.57 (s, 1H, NH), 7.36 (dd, *J* = 7.8, 1.5 Hz, 1H, ArH), 7.30–7.24 (m, 1H, ArH), 7.06–6.99 (m, 1H, ArH), 5.80 (s, 1H, =CH), 2.98–2.74 (m, 3H, OCH + CH_2_), 1.96 (d, *J* = 1.2 Hz, 3H, CH_3_), 1.83–1.75 (m, 2H, CH_2_), 1.30 (s, 6H, 2 × CH_3_); HR-MS (ESI) *m*/*z*: C_16_H_20_ClNO_2_ [M + H]^+^, Calcd. 294.1255; Found 294.1255.

*(Z)-N-(4-Methylphenyl)-5-(3,3-dimethyloxiran-2-yl)-3-methylpent-2-enamide* (**6D**), brown oil, yield 69%. ^1^H-NMR (300 MHz, CDCl_3_) δ: 7.92 (s, 1H, NH), 7.43 (d, *J* = 8.1 Hz, 2H, ArH), 7.07 (d, *J* = 8.1 Hz, 2H, ArH), 5.75 (s, 1H, =CH), 2.94–2.70 (m, 3H, OCH + CH_2_), 2.28 (s, 3H, CH_3_), 1.86 (d, *J* = 1.2 Hz, 3H, CH_3_), 1.83–1.71 (m, 2H, CH_2_), 1.28 (s, 3H, CH_3_), 1.27 (s, 3H, CH_3_); HR-MS (ESI) *m*/*z*: C_17_H_23_NO_2_ [M + H]^+^, Calcd. 274.1802; Found 274.1798.

*(Z)-N-Phenyl-5-(3,3-dimethyloxiran-2-yl)-N,3-dimethylpent-2-enamide* (**6E**), brown oil, yield 65%. ^1^H-NMR (300 MHz, CDCl_3_) δ: 7.41–7.35 (m, 2H, ArH), 7.31–7.28 (m, 1H, ArH), 7.18–7.14 (m, 2H, ArH), 5.51 (s, 1H, =CH), 3.31 (s, 3H, NCH_3_), 2.87–2.65 (m, 3H, OCH + CH_2_), 1.77–1.70 (m, 5H, CH_2_ + CH_3_), 1.32 (s, 3H, CH_3_), 1.30 (s, 3H, CH_3_); HR-MS (ESI) *m*/*z*: C_17_H_23_NO_2_ [M + H]^+^, Calcd. 274.1801; Found 274.1794.

*(Z)-N-Benzyl-5-(3,3-dimethyloxiran-2-yl)-3-methylpent-2-enamide* (**6F**), yellow oil, yield 71%. ^1^H-NMR (300 MHz, CDCl_3_) δ: 7.35–7.26 (m, 5H, ArH), 5.78 (br.s, 1H, NH), 5.61 (s, 1H, =CH), 4.46 (d, *J* = 6.0 Hz, 2H, ArCH_2_), 2.92–2.70 (m, 3H, OCH + CH_2_), 1.86 (d, *J* = 1.2 Hz, 3H, CH_3_), 1.78–1.69 (m, 2H, CH_2_), 1.28 (s, 3H, CH_3_), 1.27 (s, 3H, CH_3_); HR-MS (ESI) *m*/*z*: C_17_H_23_NO_2_ [M + H]^+^, Calcd. 274.1802; Found 274.1800.

*(Z)-N-(4-Fluorobenzyl)-5-(3,3-dimethyloxiran-2-yl)-3-methylpent-2-enamide* (**6G**), yellow oil, yield 72%. ^1^H-NMR (300 MHz, CDCl_3_) δ: 7.29–7.21 (m, 2H, ArH), 7.02–6.96 (m, 2H, ArH), 6.09 (br.s, 1H, NH), 5.62 (s, 1H, =CH), 4.40 (d, *J* = 5.8 Hz, 2H, ArCH_2_), 2.93–2.66 (m, 3H, OCH + CH_2_), 1.85 (d, *J* = 1.2 Hz, 3H, CH_3_), 1.77–1.68 (m, 2H, CH_2_), 1.27 (s, 3H, CH_3_), 1.26 (s, 3H, CH_3_); HR-MS (ESI) *m*/*z*: C_17_H_22_FNO_2_ [M + H]^+^, Calcd. 292.1707; Found 292.1707.

*(Z)-5-(3,3-Dimethyloxiran-2-yl)-3-methyl-1-morpholinopent-2-en-1-one* (**6H**), yellow oil, yield 64%. ^1^H-NMR (300 MHz, CDCl_3_) δ: 5.82 (s, 1H, =CH), 3.66 (br, 6H, 3 × CH_2_), 3.51 (br, 2H, CH_2_), 2.76 (t, *J* = 6.3 Hz, 1H, OCH), 2.49 (t, *J* = 7.8 Hz, 2H, CH_2_), 1.87 (d, *J* = 1.2 Hz, 3H, CH_3_), 1.75–1.68 (m, 2H, CH_2_), 1.30 (s, 3H, CH_3_), 1.28 (s, 3H, CH_3_); HR-MS (ESI) *m*/*z*: C_14_H_23_NO_3_ [M + H]^+^, Calcd. 254.1751; Found 254.1747.

*(Z)-5-(3,3-Dimethyloxiran-2-yl)-3-methyl-1-(pyrrolidin-1-yl)pent-2-en-1-one* (**6I**), yellow oil, yield 65%. ^1^H-NMR (300 MHz, CDCl_3_) δ: 5.84 (s, 1H, =CH), 3.51 (t, *J* = 6.6 Hz, 2H, NCH_2_), 3.44 (t, *J* = 6.6 Hz, 2H, NCH_2_), 2.72 (t, *J* = 6.3 Hz, 1H, OCH), 2.27–2.21 (m, 2H, CH_2_), 1.86 (d, *J* = 1.2 Hz, 3H, CH_3_), 1.71–1.65 (m, 6H, 3 × CH_2_), 1.31 (s, 3H, CH_3_), 1.28 (s, 3H, CH_3_); HR-MS (ESI) *m*/*z*: C_14_H_23_NO_2_ [M + H]^+^, Calcd. 238.1802; Found 238.1801.

*(Z)-N-Isopropyl-5-(3,3-dimethyloxiran-2-yl)-3-methylpent-2-enamide* (**6J**), yellow oil, yield 79%. ^1^H-NMR (300 MHz, CDCl_3_) δ: 5.55 (s, 1H, =CH), 5.35 (br.s, 1H, NH), 4.14–4.06 (m, 1H, NCH), 2.90–2.63 (m, 3H, OCH + CH_2_), 1.84 (d, *J* = 1.2 Hz, 3H, CH_3_), 1.82–1.70 (m, 2H, CH_2_), 1.38 (s, 3H, CH_3_), 1.36 (s, 3H, CH_3_), 1.15 (d, *J* = 6.5 Hz, 6H, 2 × CH_3_); HR-MS (ESI) *m*/*z*: C_13_H_24_NO_2_ [M + H]^+^, Calcd. 226.1807; Found 226.1797.

### 3.3. Bioassay of Fungicidal Activity

The preliminary fungicidal activities of compounds **5**–**6** against *F. graminearum*, *R. solani*, *A. solani*, *S. sclerotiorum*, and *B. cinerea* were evaluated using methods in the references [33,34,35,36,37] by the mycelium growth rate [38]. The culture was incubated at 25 ± 0.5 °C. Procedure for inhibition rate: The stock 2000 mg/L DMSO solution of tested compounds were prepared in advance. Then hot PDA culture medium was added into a plate, added sample solution or blank DMSO to the plate and mix with PDA culture medium, made the final concentration as desired. When plate was made, put a 5 mm diameter fungus cake into the center of plate, incubated them at 25 ± 0.5 °C for 24–48 h, checked the growth status and calculated the inhibition rate according to the reference. Three replicates were performed and the mean measurements were calculated from the three replicates for each compound.

The EC_50_ values were determined from the inhibition rates of five different concentrations based on the statistics method of [39] for the compounds that had more than 70% inhibition rates. Procedure for EC_50_ determination: the inhibition rates of compounds against different fungus at five concentrations were evaluated as before. Toxicity regression equations were obtained by statistics analysis and the EC_50_ values (µM) were calculated from the regression equations with excel program. Carbendazim and Chlorothalonil were used as positive control in the mycelium growth rate test.

## 4. Conclusions

(*Z*/*E*)-3,7-dimethyl-2,6-octadienamide derivatives and their 6,7-epoxy analogues were synthesized in moderate to excellent yields in four steps with the commercially available nerol/geraniol as raw materials. All the compounds were characterized by HR-ESI-MS and ^1^H-NMR spectral data. The preliminary bioassays showed that some of these compounds, such as **5C**, **5I** and **6b** exhibit 94.0%, 93.4% and 91.5% inhibition rates against *R. solani* at the concentration of 50 µg/mL, respectively. The EC_50_ values of compounds **5f** and **5G** were 9.7 and 13.4 µM against *R. solani*, respectively, while compound **5f** had EC_50_ value of 4.3 µM against *F. graminearum*. Further syntheses and structure optimization studies on the replacement of aromatic and aliphatic amines with nitrogen-containing heterocyclic amines are in progress in our laboratory.
