# Correction: Yang et al. Synthesis and Fungicidal Activities of (*Z*/*E*)-3,7-Dimethyl-2,6-octadienamide and Its 6,7-Epoxy Analogues. *Molecules* 2015, *20*, 21023–21036

**DOI:** 10.3390/molecules31020310

**Published:** 2026-01-16

**Authors:** Mingyan Yang, Hongbo Dong, Jiazhen Jiang, Mingan Wang

**Affiliations:** Department of Applied Chemistry, China Agricultural University, Beijing 100193, China; yangmy@cau.edu.cn (M.Y.); dhb1986115@cau.edu.cn (H.D.); jiangjiazhen@cau.edu.cn (J.J.)

## Error in Scheme 2

In the original publication [1], there was a mistake in Scheme 2 as published. The reagent SO_2_Cl_2_ was an error, and should be revised as SOCl_2_. The correct Scheme 2 legend appears below. The authors state that the scientific conclusions are unaffected. This correction was approved by the Academic Editor. The original publication has also been updated.

## Figures and Tables

**Scheme 2:**
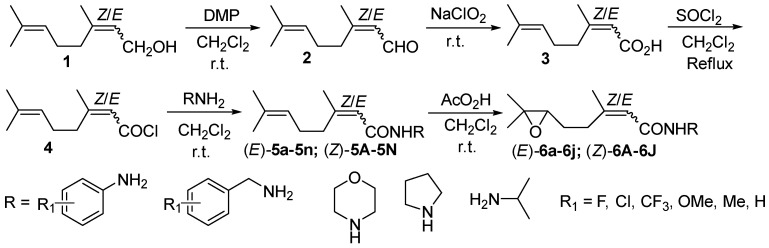
Synthetic route of (*Z*/*E*)-3,7-dimethyl-2,6-octadienamide and their 6,7-epoxy analogues.
